# Exploring the Inhibitory Potential of *M. pendans* Compounds Against *N*-Acetylglucosamine (Mur) Receptor: *In Silico* Insights Into Antibacterial Activity and Drug-Likeness

**DOI:** 10.1155/tswj/3569811

**Published:** 2024-11-30

**Authors:** Meirina Gartika, Sefren Geiner Tumilaar, Hendra Dian Adhita Dharsono, Denny Nurdin, Dikdik Kurnia

**Affiliations:** ^1^Department of Pediatric Dentistry, Faculty of Dentistry, Universitas Padjadjaran, Bandung, Indonesia; ^2^Department of Chemistry, Faculty of Mathematics and Natural Sciences, Universitas Padjadjaran, Bandung, Indonesia; ^3^Department of Conservative Dentistry, Faculty of Dentistry, Universitas Padjadjaran, Bandung, Indonesia

**Keywords:** antibacterial, *in silico*, *M*. *pendans*, MurA, MurB

## Abstract

Oral diseases are often caused by bacterial infections, making the inhibition of receptors like *N*-acetylglucosamine critical in preventing bacterial formation. The plant *Myrmecodia pendans* (*M. pendans*) is known for its diverse bioactivities and may serve as a promising source for developing new antibacterial agents. This study employs in silico methods to predict the inhibitory mechanisms, pharmacokinetics, and drug-likeness of compounds isolated from *M. pendans*. Three compounds were evaluated for their inhibitory effects on the MurA and MurB receptors using the AutoDock4 molecular docking software, with visualizations performed using the BIOVIA Discovery Studio Visualizer. The binding affinities obtained for compounds **1**, **2**, and **3** to the MurA receptor were −9.42, −9.57, and −6.84 kcal/mol, respectively, while their binding affinities to the MurB receptor were −11.25, −10.55, and −8.69 kcal/mol. These affinities were found to be stronger than those of fosfomycin (benchmark compound) but weaker than the native ligands of the respective receptors. Key amino acid residues involved in the binding to MurA were identified as Cys115 and Asp305, while Ser82 and Asn83 were noted for MurB. In the ADMET prediction and drug-likeness analysis, some compounds met the necessary criteria, whereas others did not. Although all the three compounds demonstrated strong predicted inhibitory activity against MurA and MurB receptors, the analysis suggests that Compound **2** may hold the most promise as a potential antibacterial agent, warranting further investigation.

## 1. Introduction

Dental and oral health remain neglected aspects of human wellbeing, despite the oral cavity hosting the growth of over 700 species of microorganisms capable of causing serious diseases [1, 2]. One prevalent oral health issue is dental caries, which arises from the depletion of minerals in hard tooth tissues [3, 4]. The production of lactic acid through carbohydrate fermentation and its interaction with amino acids such as serine, tyrosine, and threonine lead to the buildup of enamel on the teeth, resulting in the formation of dental caries [5, 6]. Another contributing factor is the synergy of several bacteria, such as *S. mutans* and *S. aureus*, which contribute to plaque buildup on the teeth, leading to the formation of biofilm [7–9]. Biofilm as the main mediator of dental infection is formed from initial adhesion, initial attachment, and formation of young to mature biofilms to the spreading stage [10–12]. As the prevalence of oral diseases increases, it is important to find new antibacterial agents with low toxicity, short duration of therapy, and high drug efficacy [13].

One way to treat bacterial infections, especially in tooth roots, is by inhibiting the biosynthesis of bacterial cell walls, such as in *E. faecalis*. Peptidoglycan, as the main component of the bacterial cell wall, plays a crucial role in its stability [14, 15]. These peptidoglycans are composed of polysaccharides containing *N*-acetylglucosamine (Mur) and *N*-acetylmuramic acid. *N*-acetylglucosamine enolpyruvyl transferase (MurA) and *N*-acetylglucosamine reductase (MurB) can serve as specific target receptors because they play key roles in bacterial cell wall formation. Targeting these two enzymes in the development of new antibacterial drugs is a potential strategy [16–18].

Recent studies have highlighted the significance of Mur metabolism in the pathogenesis of *E. faecalis* infections [19]. Mur is a key component of the bacterial cell wall and is also involved in various cellular processes, including biofilm formation, regulation of gene expression, and modulation of host-pathogen interactions [20]. Studies have shown that alterations in Mur metabolism can impact the expression of virulence factors in *E. faecalis*, potentially influencing its pathogenicity and antibiotic resistance [21, 22].

The development of new drugs as antibacterial compounds must continue as drug resistance continues to increase. Safety and therapeutic success are important considerations in the development of new drugs in order to minimize side effects [23]. The use of medicinal plants as an alternative source of new antibacterial drugs is gaining attention [24]. *Myrmecodia pendans* (*M. pendans*) is a plant that originates in eastern Indonesia, precisely in Papua [25]. The root extract of this plant can treat diarrhea, cancer, TB, and several other diseases [26]. The content of secondary metabolite compounds contained in this plant such as flavonoids, tannins, terpenoids, and phenolics are predicted to be used as antibacterial, antioxidant, and anticancer agents [27].

Structure-based drug design (SBDD) approach is one of the in silico techniques that can accelerate the discovery of new drug compounds predicted to be antibacterial [28, 29]. Molecular docking is one of the methods that can predict the binding of ligands or new compounds to target enzymes [30–32]. Despite the advancements provided by in silico techniques, there remains a notable gap in our understanding of how Mur metabolism specifically influences the expression of virulence factors in *E. faecalis* [33]. While our study employs computational methods such as molecular docking, absorption, distribution, metabolism, excretion, and toxicity (ADMET) prediction, and drug-likeness analysis to explore the potential of Mur-targeting compounds from *M. pendans* as antibacterial agents, it is important to acknowledge that these findings are preliminary and do not fully elucidate the underlying mechanisms of Mur metabolism in bacterial virulence. Consequently, while our research aims to identify promising candidates for further experimental validation, any assertions regarding the development of novel therapeutic strategies targeting Mur metabolism should be approached with caution. Future studies will be necessary to bridge the existing gaps and establish a clearer understanding of the relationship between Mur metabolism and virulence factor expression in *E. faecalis* infections.

## 2. Materials and Methods

The molecular docking analysis in this study followed the method of Tumilaar et al. [34]. The detailed description of materials and methods in this study is as follows.

### 2.1. Receptor Preparation and Ramachandran Plot Analysis

The RCSB Protein Data Bank (https://www.rcsb.org/) was used to select target receptors. The 3D structures of MurA (PDB ID: 1UAE) [35], which contains the native ligand uridine-diphosphate-*N*-acetylglucosamine (UD1), and MurB (PDB ID: 1HSK) [36], which contains the native ligand flavin-adenine dinucleotide (FAD), were selected as antibacterial target receptors.

The receptors downloaded from the Protein Data Bank are opened in the BIOVIA Discovery Studio Visualizer 2020 application. Water molecules were removed, and the receptor was prepared for docking using AutoDockTools, following established protocols.

The analysis of the Ramachandran plot is conducted online using PROCHECK by accessing the website (https://saves.mbi.ucla.edu) [37].

### 2.2. Ligand Preparation

**Figure 1:**
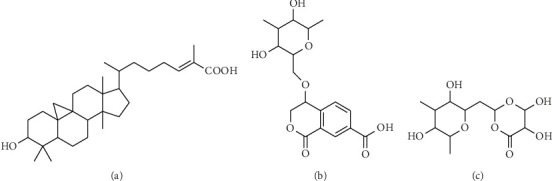
Structure of Compound **1** (a), Compound **2** (b), and Compound **3** (c) [20].

The structures of the compounds successfully isolated from *M. pendans* were used as ligands, and their 3D structures were drawn using ChemDraw Professional 16.0 and Chem3D 16.0 programs. Fosfomycin, used as a benchmark compound, was downloaded from the PubChem online web server (https://pubchem.ncbi.nlm.nih.gov/) with CID 446987 [38]. The downloaded molecules were converted to .pdb format using the OpenBabel software [39] (Figure 1).

### 2.3. Receptor-Ligand Docking and Visualization

Docking simulations were executed using AutoDock4, utilizing the AutoGrid and AutoDock commands for the parameter setup and docking process [40]. The binding affinities and interactions of the ligands with the target receptors were analyzed [41]. The molecular docking results are visualized using the BIOVIA Discovery Studio Visualizer 2020 application.

### 2.4. Drug-Likeness (RO5) and ADMET Analysis

The ProTox-II, Prediction of Toxicity of Chemicals online web server (https://tox-new.charite.de/protox_II/) [42], is utilized to predict the drug-likeness RO5 and toxicity of a molecule, while ADME prediction is performed using the pkCSM online web server (https://biosig.unimelb.edu.au/pkcsm/) [43].

## 3. Results

**Figure 2:**
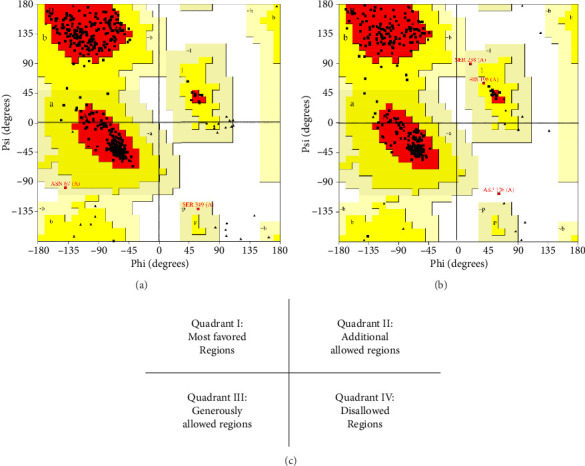
The Ramachandran plots of the receptors *N*-acetylglucosamine enolpyruvyl transferase (MurA) (PDB ID 1UAE) (a), *N*-acetylglucosamine reductase (MurB) (PDB ID 1HSK) (b), and the division of quadrants on the Ramachandran plot (c).

The receptors used in the study are the enzyme structures MurA with PDB ID 1UAE and MurB with PDB ID 1HSK, obtained from the Protein Data Bank website. These receptors were analyzed using Ramachandran plots (Figure 2). The Ramachandran plot is one of the tools used to validate receptors or proteins. It consists of four different quadrants: the most favored regions (quadrant I), additional allowed regions (quadrant II), generously allowed regions (quadrant III), and disallowed regions (quadrant IV). The Ramachandran plot is a scatter plot between phi (*ϕ*) and psi (*ψ*), reflecting the allowed areas of conformational space available for the protein chain [44].

Table 1 displays the binding affinity of the ligands to the receptors. The strength of ligand binding to the receptor is indicated by the ligand binding affinity. MurA and MurB have native ligands with binding affinities of −11.21 and −14.96 kcal/mol, respectively. In this study, fosfomycin was utilized as a benchmark compound docked to MurA and MurB receptors with binding affinities of −5.33 and −5.46 kcal/mol, respectively. At the MurA receptor, compounds **1**, **2**, and **3** are docked with binding affinities of −9.42, −9.57, and −6.84 kcal/mol, respectively. Meanwhile, compounds **1**, **2**, and **3** docked to the MurB receptor have binding affinities of −11.25, −10.55, and −8.69 kcal/mol, respectively.

In this study, the inhibition constant value of each ligand was calculated. The native ligand, fosfomycin, and compounds **1**, **2**, and **3** docked to the MurA receptor have inhibition constant (Ki) values of 6.06 nM, 124.11 *μ*M, 124.97 nM, 95.95 nM, and 9.64 *μ*M, respectively. When docked to the MurB receptor, each of these ligands has inhibition constant (Ki) values of 10.83 pM, 99.72 *μ*M, 5.65 nM, 18.42 nM, and 428.15 nM.

The interaction of amino acid residues involved in the MurA and MurB receptors occurs at ring A with the amino acid residues tryptophan (Trp), phenylalanine (Phe), alanine (Ala), glycine (Gly), asparagine (Asn), histidine (His), arginine (Arg), cysteine (Cys), valine (Val), glutamic acid (Glu), serine (Ser), proline (Pro), aspartic acid (Asp), tyrosine (Tyr), and leucine (Leu). Each of these amino acid residues forms hydrogen bonds as shown in Tables 2 and 3.

**Figure 3:**
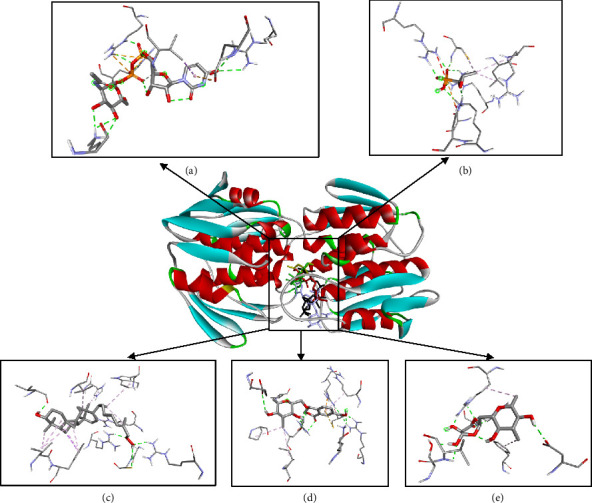
Molecular interaction area of native ligand (a), fosfomycin (b), Compound **1** (c), Compound **2** (d), and Compound **3** (e) against MurA receptor [33].

**Figure 4:**
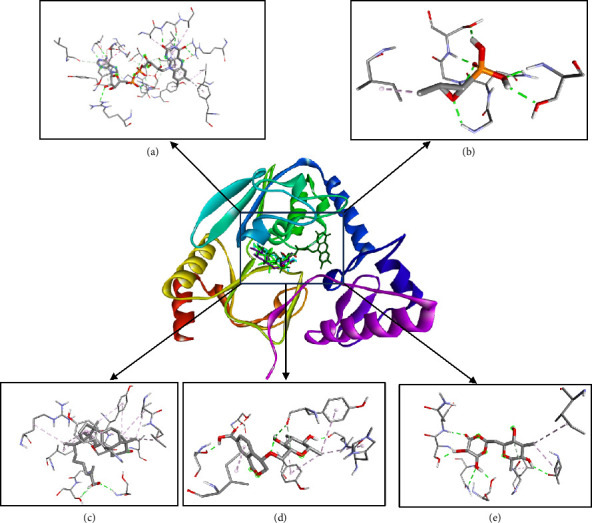
Molecular interaction area of native ligand (a), fosfomycin (b), Compound **1** (c), Compound **2** (d), and Compound **3** (e) against MurB receptor [33].

**Figure 5:**
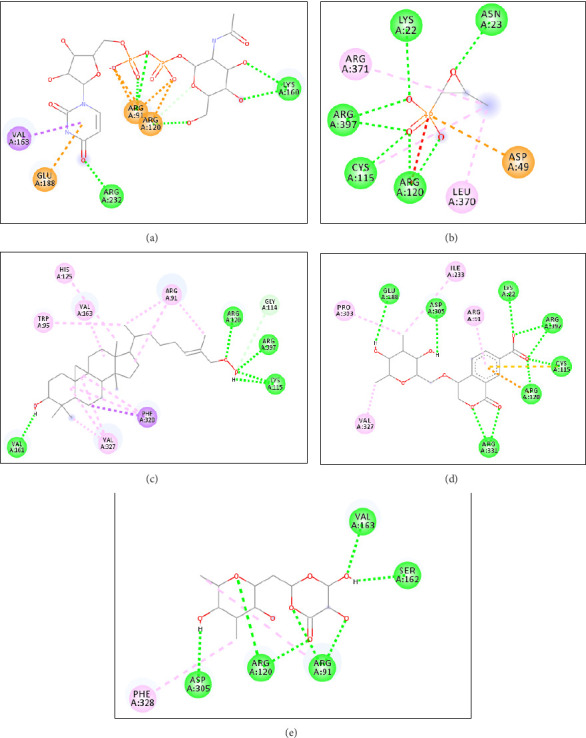
2D Conformation of amino acid residues of native ligand (a), fosfomycin (b), Compound **1** (c), Compound **2** (d), and Compound **3** (e) to MurA receptor [33].

**Figure 6:**
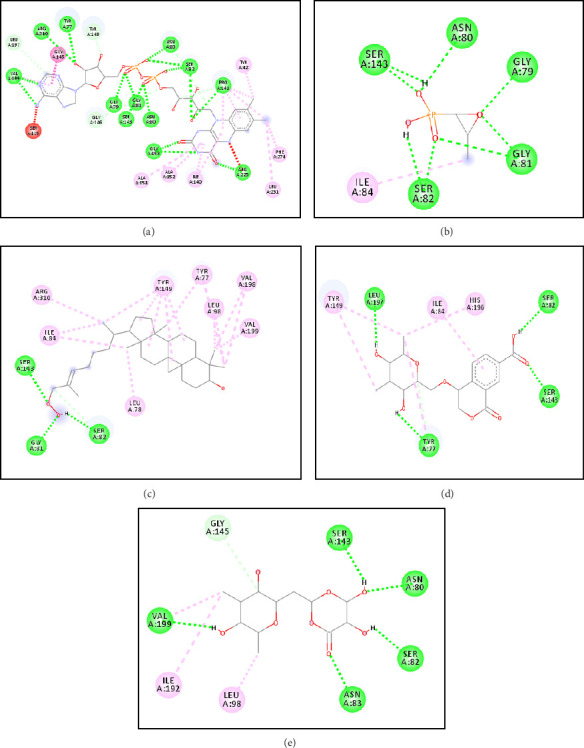
2D Conformation of amino acid residues of native ligand (a), fosfomycin (b), Compound **1** (C), Compound **2** (d), and Compound **3** (e) to MurB receptor [33].

The 3D visualizations of ligand and receptor binding can be seen in Figures 3 and 4. The grid box area for ligands docking to the MurA receptor is located at the *x*-axis = 44.50, *y*-axis = 20.97, and *z*-axis = 39.81. Meanwhile, the grid box area for ligands docking to the MurB receptor is located at *x*-axis = 179.52, *y*-axis = 148.69, and *z*-axis = 163.98. The 2D visualizations of ligand and receptor binding can be seen in Figures 5 and 6.

Table 4 presents pharmacokinetic data on absorption, distribution, metabolism, excretion, and toxicity of compounds **1**, **2**, and **3**. Several absorption parameters are considered in determining drug pharmacokinetics, including water solubility and intestinal absorption. Distribution parameters include volume distribution (VDss), blood-brain barrier (BBB) permeability, and central nervous system (CNS) permeability. Metabolic parameters involve the inhibition of CYP enzymes, while excretion parameters are determined by the overall clearance value.  Table 5 displays the medication suitability analysis, which is divided into five parameters: molecular mass, hydrogen bond donor, hydrogen bond acceptor, LogP, and molar refractivity.

## 4. Discussion

Molecular docking is one of the in silico techniques used to predict the mechanism of ligand inhibition against receptors [45, 46]. The receptors selected in this study are Mur receptors because they play a key role in bacterial growth. Inhibiting bacterial growth is one way to inhibit the bacterial cell wall, which contains peptidoglycan, a key component of this receptor. If this receptor is inhibited, bacteria cannot grow rapidly [47, 48].

There are two types of Mur receptors, namely, MurA and MurB. Receptor analysis is validated using Ramachandran diagrams based on the protein's secondary structure. A receptor structure is considered good if the number of nonglycine amino acid residues found in the favored regions is more than 90%, and the amino acid residues in quadrant IV are less than 20% [49]. The main chain of the N-C*α* and C*α*-C bonds in a polypeptide is relatively free to rotate, and these rotations are, respectively, called the phi and psi torsion angles [50]. The Ramachandran diagram represents a polypeptide systematically varying phi and psi angles in order to obtain stable conformations. Conformational structures are analyzed through contact plots between atoms. Phi represents the *x*-axis, and psi represents the *y*-axis of the amino acid residues in a protein structure [51]. The analysis results based on the Ramachandran plot show that the amino acid residues of the MurA receptor (1UAE) are in quadrant I at 93.6%, quadrant II at 5.8%, quadrant III at 0.6%, and quadrant IV at 0.0%. Meanwhile, for the MurB receptor (1HSK), it is in quadrant I at 90.8%, quadrant II at 8.0%, quadrant III at 0.8%, and quadrant IV at 0.4% (Figure 2). These results indicate that the structures of both receptors have very good quality for use as receptors in molecular binding because the amino acid residues in quadrant IV are less than 20% [52]. Furthermore, the amino acid residues of both receptors predominantly reside in quadrant I and quadrant II (Figure 2).

The predicted active site area of MurA is found at amino acid residues Arg93, Cys115, Asp305, and Val327 [53, 54]. In this study, Compound **1** forms a hydrogen bond with one active site, Cys115. Compound **2** forms hydrogen bonds with two active sites, namely Cys115 and Asp305, while Compound **3** binds to one active site, Asp305. The number of hydrogen bonds with the active site residues of the receptor correlates with the strength of the ligand-receptor bonds [55] (Figures 3 and 5). The active site of MurB is located at amino acid residues Ser82, Asn83, Arg188, and Arg310 [17, 56]. Compounds **1** and **2** form hydrogen bonds with one amino acid residue, Ser82, while Compound **3** forms hydrogen bonds with two amino acid residues, Ser82 and Asn83 (Figures 4 and 6).

In addition to the amino acid residues involved, the binding affinity value must also be considered. Binding affinity refers to the strength of the binding interaction between a small biomolecule ligand and its receptor (large biomolecule) [57, 58]. The smaller the binding affinity value, the stronger the inhibition of the ligand on the receptor [59]. Compounds **1**, **2**, and **3** binding to the MurA receptor have binding affinities of −9.42, −9.57, and −6.84 kcal/mol, respectively. These binding affinity values are lower than that of fosfomycin, the benchmark compound, which only has a binding affinity of −5.33 kcal/mol. Fosfomycin was selected as a benchmark compound because it has been demonstrated to be a competitive inhibitor of the Mur enzyme [60]. At the MurB receptor, Compounds **1**, **2**, and **3** exhibit binding affinities of −11.25, −10.55, and −8.69 kcal/mol, respectively. Again, these values are lower than that of fosfomycin, the benchmark compound, which only has a binding affinity of −5.46 kcal/mol. Although these values are not smaller than those of the native ligand, they indicate that each docked ligand shows strong potential to inhibit the Mur receptor. Furthermore, considering the interaction of the amino acid residues involved and the binding affinity value as a parameter of ligand inhibition against the receptor, Compound **2** may hold the most promise as a potential antibacterial agent, warranting further investigation.

The next parameter to consider is the Ki value. The Ki value depends on the binding affinity of the ligand. The greater the binding affinity to the receptor, the smaller the Ki value of the ligand [61]. This Ki value indicates the ability of a compound to inhibit the receptor. At the MurA receptor, Compounds **1** and **2** have Ki values of 124.97 nM and 95.95 nM, respectively. These values are smaller than the Ki value of Compound **3**, which has a Ki value of 9.64 *μ*M. The Ki values of Compounds **1**, **2**, and **3** are 5.65, 18.42, and 428.15 nM, respectively.

The next consideration in computational studies is predicting drug pharmacokinetics, such as ADMET. Compounds isolated from plants must meet good ADMET criteria to be considered as new drug candidates [62, 63]. Conducting ADMET considerations computationally is more efficient and cost-effective [64].

In terms of absorption, two parameters need to be considered: water solubility and intestinal absorption [65]. The compounds solubility must fall within the range of values greater than −5 and smaller than 0 [66]. Based on Table 4, three compounds fall within the appropriate range. Regarding intestinal absorption, a value above 80% is desirable [67]. Compounds **1** and **2** have intestinal absorption values above 80%, at 95.61% and 81.78%, respectively. However, Compound **3** is below 80%, with a predicted value of 46.78%.

Moving to the drug distribution phase, three parameters are crucial: volume distribution, BBB permeability, and CNS permeability [68, 69]. The distribution volume of a drug in the blood should range between 0.5 and 3 L/kg [70]. Compounds **2** and **3** meet this requirement, while Compound **1** does not. A good drug candidate should have limited penetration of the CNS and BBB [71]. There are three categories for drug absorption into the CNS and BBB. A value greater than two indicates high absorption, between 0.1 and two indicates moderate absorption, and less than 0.1 indicates low absorption [72]. These three compounds fall into the low absorption category, with values less than 0.1.

Cytochrome P450 (CYP450) is an enzyme crucial in the digestive system and should not be inhibited [73]. The three compounds showed no inhibition of this enzyme. Regarding excretion parameters, higher total clearance values indicate faster molecule excretion [74]. Toxicity prediction can be conducted through computational techniques, with toxicity assessed by the lethal dose (LD) value. LD50 greater than 5000 mg/kg is categorized as nontoxic, while LD50 less than 5000 mg/kg is considered a potentially dangerous drug if ingested [75, 76]. Compounds **2** and **3** are categorized as nonhazardous, while Compound **1** is predicted to be dangerous if taken orally.

A compound should also undergo drug-likeness analysis by following Lipinski's rule of five. A compound can be considered a new oral drug candidate if it does not exceed one violation of this rule [77, 78].  Table 5 shows that Compounds **2** and **3** meet this criterion, while Compound **1** still requires consideration as it exceeds one violation of this rule.

Our findings underscore the importance of Mur metabolism in *E. faecalis* pathogenesis, aligning with previous studies demonstrating its critical role in bacterial virulence [79, 80]. The significant impact of Mur metabolism on the expression of virulence factors highlights its potential as a target for antibacterial intervention strategies [81]. Furthermore, our results are consistent with recent research showing the effectiveness of targeting Mur metabolism in combating bacterial infections. For example, inhibiting Mur utilization impairs biofilm formation and attenuates *E. faecalis* virulence in a murine model of infection [82]. Similarly, Naclerio et al. [83] reported that Mur metabolism inhibitors enhance the efficacy of antibiotics against multidrug-resistant *E. faecalis* strains. In addition, to its direct effects on virulence factor expression, Mur metabolism may also influence *E. faecalis* antibiotic resistance mechanisms. Our study contributes to this body of literature by identifying potential Mur-targeting compounds from *M. pendans* with promising antibacterial activity against *E. faecalis*. However, it is essential to acknowledge the limitations of our in silico approach and the need for further validation through in vitro and in vivo experiments. Future research should explore the synergistic effects of Mur metabolism inhibitors with existing antibiotics and investigate their potential clinical applications in treating *E. faecalis* infections.

## 5. Conclusion

The in silico analyses conducted in this study suggest that the three compounds isolated from *M. pendans* show potential for antibacterial activity, particularly through their predicted interactions with the MurA and MurB receptors. The molecular docking results indicate that these compounds may exhibit moderate to strong binding affinities compared with the reference compound, fosfomycin. In addition, ADMET predictions and drug-likeness analyses suggest that Compound **2** demonstrates the most favorable pharmacokinetic properties and drug-likeness potential. However, these findings are preliminary and based solely on computational predictions. Further experimental validation, including in vitro and in vivo studies, is required to confirm the actual antibacterial efficacy of these compounds and their mechanisms of action. Ultimately, our goal is to contribute to the discovery and development of effective antibacterial agents to address the growing challenge of antibiotic resistance and infectious diseases.

## Figures and Tables

**Table 1:** Prediction of antibacterial activity against MurA and MurB receptors.

Receptor	Ligand	Binding affinity (Kcal/mol)	Inhibition constant (Ki)
MurA (1UAE)	Native	−11.21	6.06 nM
	Fosfomycin	−5.33	124.11 µM
	Compound **1**	−9.42	124.97 nM
	Compound **2**	−9.57	95.95 nM
	Compound **3**	−6.84	9.64 µM

MurB (1HSK)	Native	−14.96	10.83 pM
	Fosfomycin	−5.46	99.72 µM
	Compound **1**	−11.25	5.65 nM
	Compound **2**	−10.55	18.42 nM
	Compound **3**	−8.69	428.15 nM

**Table 2:** Hydrogen, hydrophobic, and electrostatic interactions between ligands and MurA receptor.

Ligand	Type of interaction	Interacting residues with chain A
Native	Conventional hydrogen bond	Arg91, Arg120, Lys160, Arg232
	Carbon hydrogen bond	Arg120
	Pi-Anion	Arg91, Arg120, Glu188
	Pi-Sigma	Val163

Fosfomycin	Conventional hydrogen bond	Lys22, Asn23, Cys115, Arg120, Arg397
	Alkyl/Pi-Alkyl	Cys115, Leu370, Arg371

Compound **1**	Conventional hydrogen bond	Cys115, Arg120, Val161, Arg397
	Carbon hydrogen bond	Gly114
	Alkyl/Pi-Alkyl	Arg91, Trp95, His125, Val163, Val327, Phe328
	Pi-Sigma	Phe328

Compound **2**	Conventional hydrogen bond	Lys22, Cys115, Arg120, Glu188, Asp305, Arg331, Arg397
	Alkyl/Pi-Alkyl	Arg91, Ile233, Pro303, Val327
	Pi-Cation	Arg120
	Pi-Sulfur	Cys115

Compound **3**	Conventional hydrogen bond	Arg91, Arg120, Ser162, Val163, Asp305
	Alkyl/Pi-Alkyl	Arg91, Phe328

**Table 3:** Hydrogen, hydrophobic, electrostatic interactions ligands and MurB receptor.

Ligand	Type of interaction	Interacting residues with chain A
Native	Conventional hydrogen bond	Tyr77, Gly79, Asn80, Gly81, Ser82, Asn83, Pro141, Ser143, Gly153, Val199, Arg225, Arg310
	Carbon hydrogen bond	Asn83, Pro141, Gly146, Tyr149, Leu197
	Alkyl/Pi-Alkyl	Tyr42, Ile140, Pro141, Ala152, Ala154, Leu231, Phe274
	Amide-Pi Stacked	Gly145

Fosfomycin	Conventional hydrogen bond	Gly79, Asn80, Gly81, Ser82, Ser143
	Alkyl/Pi-Alkyl	Ile84

Compound **1**	Conventional hydrogen bond	Gly81, Ser82, Ser143
	Carbon hydrogen bond	Ser82
	Alkyl/Pi-Alkyl	Tyr77, Leu78, Ile84, Leu98, Tyr149, Val198, Val199, Arg310

Compound **2**	Conventional hydrogen bond	Tyr77, Ser82, Ser143, Leu197
	Carbon hydrogen bond	Tyr77
	Alkyl/Pi-Alkyl	Tyr77, Ile84, Tyr149, His196

Compound **3**	Conventional hydrogen bond	Asn80, Ser82, Asn83, Ser143, Val199
	Carbon hydrogen bond	Gly145
	Alkyl/Pi-Alkyl	Leu98, Ile192, Val199

**Table 4:** ADMET prediction of Compounds **1**, **2**, and **3**.

Properties	Parameters	Ligands		
		Compound 1	Compound 2	Compound 3
Absorption	Water solubility (log mol/L)	−4.479	−2.702	−1.91
	Intestinal absorption (%)	95.61	81.78	46.78

Distribution	Volume distribution (VDss) (log L/kg)	−0.943	1.074	0.356
	BBB permeability (log BB)	−0.492	−1.067	−0.599
	CNS permeability (log PS)	−1.643	−3.494	−3.594

Metabolism	Inhibitor of CYPIA2	No	No	No
	Inhibitor of CYP2C19	No	No	No
	Inhibitor of CYP2C9	No	No	No
	Inhibitor of CYP2D6	No	No	No
	Inhibitor of CYP3A4	No	No	No

Excretion	Total clearance (log ml/min/kg)	0.262	1.035	1.317

Toxicity	Lethal Dose 50% (mg/kg)	1925	60,000	8000

**Table 5:** Drug-likeness Lipinski's rule of five (RO5).

Parameters	Ligands		
	Compound 1	Compound 2	Compound 3
Molecular mass (< 500 D)	470.73	366.36	292.28
H-bond donors (< 5)	2	2	4
H-bond acceptors (< 10)	53	30	28
LogP (< 5)	7.62	0.76	−1.9
Molar refractivity (40–130)	141.72	88.37	63.67
Violation	3	1	1
Drug-likeness	No	Yes	Yes

## Data Availability

All data used to support the findings of this study are included within the article.
